# Whither Midwifery?

**Published:** 1983-04

**Authors:** Gordon M. Stirrat

**Affiliations:** Professor of Obstetrics & Gynaecology, University of Bristol


### Whither Midwifery?

**Gordon M. Stirrat, M.A., M.D., F.R.C.O.G.**
Professor of Obstetrics & Gynaecology, University of Bristol

The title given to this evening's lecture is purposely a broad one and is designed to allow me, the speaker, to say exactly what I wish, about anything I wish, as long as it bears some relation to the topic. Indeed human nature being what it is – it is likely that the more obscure my allusions to the entitled topic the more erudite I will be deemed to be. Erudition is not, however, an attribute I have been accused of possessing: my most vivid remembrance of that was the cry of my form and classics master in the Glasgow school I shared (not simultaneously) with John Buchan and described by him in 'Memory Holds the Door' as 'an ancient grammar school on the south side of the river'. 'Stirrat' cried Chunky Cowan, the aforesaid form master, 'Stirrat, you are a fool – you may be a clever fool but you are still a fool.'

Ah well, since it is not within me to be erudite, I'm afraid I will have to address the topic entrusted to me directly and, I hope, comprehensibly. I trust, however, that your comprehension does not have that disturbing quality about it which I experienced when I was taken to see the original film version of 'MASH'. That is that I thought I understood most of the humour but rather wished I did not.

### MIDWIFERY v OBSTETRICS

In discussing the question of 'Whither Midwifery?' I possess the advantage (not a unique advantage, I hasten to add, for there are others in this audience from the same or similar – dare I say lesser – stables) of having been nurtured in a university 'Department of Midwifery' – not of Obstetrics, under the tutelage of Ian Donald. 'What is the difference?' I hear you ask—they even mean roughly the same! (Mid – with; obstet – to stand alongside). I do believe, however, that there is a fundamental difference between the two words. To illustrate it, I would ask you to do something very simple. Namely consider the contrasting images produced in your mind by the two words 'midwife' and 'obstetrician'. The first conveys a reassuring sense of the normal aspects of childbirth: the second brings with it overtones of pathology: of 'mothers' becoming 'patients'.

But before you jump to the conclusion that I am, in obstetric terms, one of those 'trendy-lefties' espousing the fads and fashions of the moment, let me reassure you that I acknowledge that normality can only be fully appreciated when one has the skills and resources available to deal with the abnormal.

I would ask you, therefore, to return to those mental images and the model of my alma mater. Obstetrics is, I would suggest, something one practises in the context of Midwifery, rather than Midwifery in the context of Obstetrics. It is a source of sadness to me that the latter seems to have become increasingly true over the last decade. I will return to that later.

### WHITHER MIDWIFERY?

If, as the title suggests, I am required to make predictions as to future trends and developments, I am reminded of the many confident predictions of yesteryear which have turned to dust and ashes almost as soon as they were uttered. Who was it, for example, who called income tax 'merely a temporary measure'? He, I am sure, is turning in his grave so fast that one expects him to emerge from it like a ballistic missile fired from its silo! (And I'm sure we all know which target we would wish him to aim for.)

However, in order that we may better look forward, I think it would be useful if we first took a look back: and because my conjectures for the future will not take us beyond the end of the century, I think it would be fitting if we looked back for a similar time span, namely 20 years. This is doubly fitting because it is probably fairly close to the mean (or perhaps median) experience of those present this evening.

The first decade of the period over which we are going to look back was the time of the highest postwar birthrate of 18 to 19/1000 home population, compared to about 12 today. The beginning of that decade (1960) saw a Maternal Mortality Rate of 0.31 and a Perinatal Mortality Rate of 33/1000 total births. In the middle of the period which we are reviewing (i.e. c. 1970) the Maternal Mortality Rate had fallen to 0.14 and the Perinatal Mortality Rate to 23/1000 total births. Today the respective rates are $<0.1$ and 15.

Much of what I wish to say about the previous 20 years is encapsulated by those rather stark figures and many of the vivid images which linger in the minds of those of us involved in caring for pregnant women over this period relate to events which are, perhaps, reflected (or could so easily have been) however insignificantly within those statistics.

Lecture given to the Northern Counties Medical Society at Newcastle-upon-Tyne, and to the Cossham Medical Society in December 1982.

In Glasgow those were still the days of home confinement in grey tenements because, for some women, there was nowhere else to go. Some of the experiences which are most deeply seared on my conscious mind are of hectic flying squad calls to one part of the city or another, to try to retrieve some calamity to mother or baby or both, using all the experience gained from 6 months' obstetrics. They were still the days of the 'grande multipara' and few of them could remember what menstruation was, let alone the date of the L.M.P.

Who can forget the labour wards in some corner of which there was always some poor primigravida with a malnourished android pelvis grinding her way through an O.P. Labour. Analgesia gradually progressed through Pethidine to Omnopon and Heroin.

Now that those memories have been revived, what other pictures are coming to your mind? Eclampsia and its control by rectal Avertin? Catastrophic placental abruptions with uncontrollable coagulation defects? The plight of the diabetic or Rhesus isoimmunised woman? The young girl dying in renal failure thanks to the ministrations of the criminal abortionist? One of my other memories from this time is that obstetric success or failure tended to be judged in terms of whether the baby was alive or dead. If it was born alive – that was success. Little thought was given to the *quality* of the child's subsequent life, not because we were unconcerned, but because there was little we could do about it anyway.

Of course, there are many other *happy* memories, and what I have said must not be taken as representative of childbirth as a whole at that time. But I felt it appropriate to highlight problem areas, to show, firstly, that at that time we seemed very much to be controlled by events rather than controlling them, and secondly to remind us that dramatic changes have occurred within these last two decades.

What has brought about these changes? The first and foremost influence has, undoubtedly, been a dramatic drop in the birthrate, and the second – better social conditions. I do not think I can overemphasise to you the importance of the former. This can be illustrated to you by taking the City of Glasgow as an example where social conditions improved for most families at a time when the traditional sources of wealth were at the beginning of a rapid decline. For example, as a House Officer I could stand on the top of the new Queen Mother's Hospital in Glasgow and look down the Clyde. The docks were still full of ships being loaded and unloaded. The sounds of hammering, riveting and welding rose from every shipyard where yet another cargo ship or tanker was gradually taking shape. Today, the wharves are empty; the shipyards are silent: the towering cranes long since gone. What was it, then, that allowed social conditions to improve despite all this? Nothing more nor less than the lessening of the burden of childbearing on the womenfolk. Backed up, I hasten to add, by the benefit of the National Health Service and the Welfare State.

In relation to the practice of Midwifery, the reduction in the birthrate brought much-needed room to move and time to think. The old technology was dying, the new beginning to bear fruit.

New, powerful, pharmacological agents became available to us and our patients. Although the decline in birthrate began before the introduction of the combined oral contraceptive pill, it was the general acceptance of the latter which accelerated things. The routine use of ergometrine allowed better control of post-partum blood loss. The production of synthetic oxytocin allowed the obstetrician to proceed to induce labour with a reasonable expectation of success. More and more new antibiotics were introduced.

Anaesthetic agents became less noxious and our anaesthetic colleagues more skilled in their use, particularly in obstetrics. Caesarean section became very much safer for the mother, to the baby's benefit.

Epidural anaesthesia was introduced into obstetrics and the horrors of the prolonged labour became manageable almost overnight. The new electronics began to make continuous observation of the fetal heart possible. The use of the wartime Asdic submarine detection equipment, transferred to industrial use in the shipyards, and observed there by Professor Donald, led to his consideration of the potential value of ultrasound in obstetric and gynaecological diagnosis. The rest is history and it is one of the most shameful episodes in medical politics in this country that a man who has contributed so much has had so little national recognition for it.

Other flashes of genius or the application of marvellously simple techniques also helped to transform things. The introduction of anti-D immunoglobulin by Sir Cyril Clarke for the prevention of Rhesus disease comes into the first category, and the partogram by Hugh Philpott and others for the graphic display of progress in labour is an example of the second.

Other changes relied on a better understanding of physiological mechanisms, particularly perhaps in developmental physiology. We, therefore, began to be aware of our perinatal responsibilities and opportunities, and neonatal paediatrics was born.

Nor were our chemical pathological colleagues far behind. New methodology, such as radioimmunoassay were increasingly improving our ability to diagnose and monitor disease processes.

The benefits from a thousand and one advances were considerable but it was not without cost! As, for example, the penalties of intervention became less, the indications for them became less clear. In consequence by the mid 1970s the incidence of induction of labour exceeded 50% in some units and reached 75% among some individual consultants.

In addition, too great a specificity and sensitivity were ascribed to the newly developed diagnostic methods: and although their introduction was undoubtedly of clinical benefit, their uncritical and universal application as THE answer to the problem in question led, in some cases, to the ascribing of pathology where none existed; and, in others, to the failure to detect it when it did.

The same relatively uncritical acceptance pertained to our use of increasingly powerful drugs. 'I don't think it will do harm, therefore it must do good' was, and is, the argument. This was despite the example of late, severe complications in the offspring of women treated with D.E.S. for a whole variety of pregnancy problems.

In addition, the increasing application of 'machinery', biochemistry and pharmacology to the management of the pregnant woman was lessening the role of the midwife and making her subservient to the obstetricians.

So much for some of the credits and debits in midwifery over the past 20 years. What of the future and what lessons can be learnt from the past?

### LOOKING FORWARD

In looking forward it is important that we try to differentiate between what we want to happen, what should happen (which are not necessarily synonymous) and what will happen. In this context I am reminded of my boyhood hero 'Dan Dare' of the Eagle comic. In my recollection of his victories over the Treens and the Mekon, he and Digby (who looked incredibly like my aforementioned form master Chunky Cowan, by the way) always returned to their base in London. But it was a London which none of us would recognise. Not one of the familiar land marks remained. All was new, and gleaming: automated and perfect. So many predictions regarding the future are like that. Nothing familiar remains. They are flights of fancy which bear little resemblance to reality.

There are, of course, some things which are *not* going to happen. For example, the dramatic fall in maternal and perinatal mortality will not continue over the next decade. There is likely to be *some* fall in maternal mortality, but it will be slight given that in a free society (and I am assuming that we will continue to live in a free society), people will remain at liberty to be irresponsible: and given that the residual part of any problem is the hardest to solve even if unlimited resources are available (which they are not).

In relation to perinatal mortality, I believe that it will *rise* over the next decade. It will do so for several reasons: firstly, it will be redefined to include pregnancies ending after 20 (or perhaps 22) weeks. Secondly, as our population becomes older, more health service resources will be spent on the elderly and less on maternity services. Thirdly, our 'consumers' perception of risk is different from ours and we are not to be allowed to forget the unnecessary interventions of the 1970s. An increasing number of at risk women will demand 'nice experiences' rather than 'clinical safety' with the result that some babies will die or be harmed unnecessarily. I fear, however, that the blame for that will be laid at our door. Fourthly, given that with current knowledge and practice we can do little to influence the two main causes of perinatal death (congenital anomaly and pre-term delivery) the PNM rate in some centres is already almost at an irreducible minimum.

However, by the end of the century, we will, I believe, understand these problems far better. As a result there is a real prospect that we will be able to prevent the majority and treat some of the remainder of them. The perinatal mortality will, therefore, begin to fall again.

Our understanding of basic physiological and pathological mechanisms will continue to increase, thanks to new techniques which have and will become available. In my own field I have in mind such things as monoclonal antibody technology, and I am sure you can think of others. We will understand a great deal more about such enigmas as pre-eclampsia and our ability to explore the fetal milieu will improve dramatically. Diagnostic imaging with ultrasound and, particularly, NMR backed up by computer enhancement will open exciting vistas to us.

Fetal biochemistry will become routinely available to us: firstly in labour where continuous monitoring of fetal acid-base balance will be achieved relatively soon.

Pharmacological advances will continue and, although not strictly in the realm of 'midwifery' I look forward to the day when I can stop performing hysterectomies merely for the relief of menorrhagia.

The lessons of the past remain with us, however, and I fear that mankind in general and its medically qualified variety in particular will remain as uncritical as ever. I trust that the consequences of our errors will not always be directly proportional to the power of the misplaced technology.

One over-riding consideration for which it is impossible to legislate in such an over-view is the extent to which economic stringency will affect matters. I do not find much to be complacent about in the way this, or previous, governments have handled our affairs in the Health Service or the Universities. I know that the situation in the Health Service is not rosy but the problem facing the clinical faculties in our universities is graver than is generally realised. Our already fragile ability to undertake worthwhile, basic research is seriously threatened. Having begun to feel able to control events, I fear that the next decade will be a time when once again events will be controlling us!

I am only too well aware that the views I have expressed are quite literally 'insular'. That was the way in which I interpreted my remit. It is, of course, necessary to recognise that events in what has been called the 'global village' are likely to influence us even more in the future than they have in the past.

In closing I would like to take a brief backward glance once more. Despite all I have said about changes and advances there is a great deal which has *not* changed over the years – nor should it have done!

When I am allowed to carry out a normal delivery I do so in more or less the same manner as I was taught by the midwives in the Glasgow Royal Maternity Hospital, 20 years ago, and I firmly believe the role of the midwife to be central in the care of the pregnant woman and her baby. Behind that there is a basic relationship which has not changed – the mother and her baby being *cared for* by the midwife and the doctor.

I am glad that has not changed and I hope it never does.

